# Induction therapy with ^177^Lu-DOTATATE procures long-term survival in locally advanced or oligometastatic pancreatic neuroendocrine neoplasm patients

**DOI:** 10.1007/s00259-022-05734-8

**Published:** 2022-03-01

**Authors:** Noémie S. Minczeles, Casper H. J. van Eijck, Marjon J. van Gils, Marie-Louise F. van Velthuysen, Els J. M. Nieveen van Dijkum, Richard A. Feelders, Wouter W. de Herder, Tessa Brabander, Johannes Hofland

**Affiliations:** 1 Department of Internal Medicine, Section of Endocrinology, Erasmus MC and Erasmus MC Cancer Centre, Dr. Molewaterplein 40, 3015 GD Rotterdam, The Netherlands; 2 Department of Radiology & Nuclear Medicine, Erasmus MC, Rotterdam, The Netherlands; 3 Department of Surgery, Erasmus MC, Rotterdam, The Netherlands; 4 Department of Pathology, Erasmus MC, Rotterdam, The Netherlands; 5 Department of Surgery, Cancer Centre Amsterdam, Amsterdam UMC, University of Amsterdam, Amsterdam, The Netherlands

**Keywords:** Peptide receptor radionuclide therapy, Neuroendocrine, Pancreas, Surgery

## Abstract

**Purpose:**

Peptide receptor radionuclide therapy (PRRT) with ^177^Lu-DOTATATE induces objective response in up to 57% of pancreatic neuroendocrine neoplasms (panNENs). Therefore, PRRT may comprise a downstaging option for panNEN patients who are not eligible for upfront curative surgery or are at high risk for recurrence. The aim of this study was to assess the potency of induction PRRT for locally advanced panNENs and to evaluate the effect of surgery after PRRT on overall survival (OS).

**Methods:**

Retrospective cohort study of panNEN patients treated with induction ^177^Lu-DOTATATE.

**Results:**

After PRRT, 26 out of 49 patients underwent pancreatic surgery with curative intent (PRRT + surgery). Partial objective response was obtained in 62% of the PRRT + surgery group versus 26% of the patients not undergoing panNEN surgery (PRRT-only group, *p* = 0.02). Downstaging in tumour-vessel interface was observed in 38% of all patients with at least one involved vessel. Median OS was 14.7 years (95% CI 5.9–23.6) for the PRRT + surgery group compared to 5.5 years (95% CI 4.5–6.5) for the PRRT-only group (*p* = 0.003). In the Cox proportional hazards analysis, surgery was not significantly associated with OS after propensity score adjustment with cumulative activity, performance status, tumour size after PRRT, and tumour grade. Median progression-free survival was 5.3 years (95% CI 2.4–8.1) for the PRRT + surgery group and 3.0 years (95% CI 1.6–4.4) for the PRRT-only group (*p* = 0.02).

**Conclusion:**

Early administration of PRRT followed by surgery is associated with favourable long-term outcomes in patients with locally advanced or oligometastatic panNEN and can be considered for selected patients with vascular involvement and/or increased risk of recurrence.

## Introduction

At the time of diagnosis, 38% of patients with a pancreatic neuroendocrine neoplasm (panNEN) present with local, 16% with regionally advanced, and 44% with metastatic stages of the disease [1]. Disease stage constitutes a major contributor to overall survival (OS) [1–5].

Surgery is the first treatment option for panNENs larger than 2 cm as this is the only curative treatment option [6, 7]. Local vascular involvement, adjacent organ invasion, and presence of liver metastases can prevent a curative resection due to the complexity or impossibility of a surgical resection [8, 9] and the increased recurrence risk [10–14]. Given the high percentage of patients presenting with advanced disease stages, there is an unmet need for therapeutic induction and neo-adjuvant strategies that can effectively downstage the disease to allow for less extensive curative surgical intervention and improve the long-term outcomes.

Peptide receptor radionuclide therapy (PRRT) with [^177^Lu-DOTA^0^,Tyr^3^]octreotate (^177^Lu-DOTATATE), a radiolabelled somatostatin analogue that targets mainly the type 2 somatostatin receptor (SSTR), is a registered second/third-line therapy for progressive metastatic well-differentiated gastroenteropancreatic neuroendocrine tumours. The objective response (OR) rate of ^177^Lu-DOTATATE in panNEN ranges 13–57% [15–18]. Hence, PRRT could be a treatment option with downstaging or neo-adjuvant potential.

Improved resectability of locally advanced or oligometastatic panNEN following PRRT has been described in a limited group of patients [19–21], but a detailed analysis of tumour downstaging and OS is lacking. In this study, we aim to evaluate the efficacy of PRRT to downstage locally advanced or oligometastatic panNENs and also to assess the effect of surgery after PRRT on OS.

## Patients and methods

### Patient selection

A retrospective, single-centre analysis was performed on all Dutch panNEN patients who received ^177^Lu-DOTATATE with a downstaging or neo-adjuvant intent between 2000 and 2019. Given the lack of validated criteria for resectability of panNENs, the tumours were deemed unresectable (locally advanced and/or metastatic disease) or borderline resectable according to the experienced view of the multidisciplinary team (MDT). A subgroup of these patients treated until June 2011 has been reported previously [19]. Sufficient tumour uptake on SSTR imaging (at least equal to the normal liver uptake on ^111^In-DTPA-octreotide scan or higher than the liver on ^68^ Ga-DOTATATE PET-CT) was a requirement for PRRT. Other inclusion criteria were published previously [15]. Patients’ follow-up information was updated until April 2020. The study was conducted in accordance with the recommendation of the Declaration of Helsinki and was performed after approval by our local institutional review board.

### Treatment

Four cycles of 7.4 GBq ^177^Lu-DOTATATE were administered with an interval of 6–10 weeks. In case of toxicity, the amount of radioactivity or number of cycles could be adjusted.

### Outcomes

The primary endpoint was OS, defined as the time from treatment initiation to death due to any cause. Secondary endpoints were PFS, treatment response, disease-free survival (DFS), and treatment-related adverse events. PFS was defined as the time from treatment initiation to morphological disease progression (assessed by RECIST 1.1 [22]), clinically relevant disease progression according to the treating physician or death by any cause. Treatment response was evaluated at 6 weeks, 3 months, and 6 months after the last treatment, and then every 6 months until disease progression. The DFS was calculated from the date of surgery until disease recurrence or last date of follow-up. Adverse events were scored according to CTCAE version 5.0 [23].

### Radiological assessment

An expert radiologist, uninformed about the patient’s characteristics, reassessed the baseline scan for all patients, the last scan prior to surgery for the surgical patients, and the scan with the best tumour response for the non-surgical patients for the tumour-vessel interface (TVI) and presence of nearby organ invasion and metastases. Degrees of tumour contact of the superior mesenteric artery (SMA), celiac axis (CA), common hepatic artery (CHA), superior mesenteric vein (SMV), and portal vein (PV) were determined on axial contrast-enhanced CT or MRI images before and after PRRT and were categorized as no TVI, ≤ 180° of the vessel’s circumference, and > 180° of the vessel’s circumference or occlusion/thrombus. We further categorized the vascular involvement in order of severity [24]. Relevant decrease (downstaging) or increase in TVI was counted when a respectively lower or higher involvement category was present after PRRT compared to baseline.

### Pathological assessment

The tumour grade was revised by an expert pathologist according to the WHO NEN classification 2019 [25]. Surgical resection specimens were reassessed for the percentage of viable tumour and fibrosis, presence of necrosis, percentage of SSTR2a-positive tumour cells, Ki67-index, and whether the treatment response was homogeneous or heterogeneous. Resection margins were described as R0 (complete resection) or R1 (complete macroscopic resection but microscopically tumour cells visible within 1 mm from the resection margin).

### Statistical analysis

For comparison between groups, χ^2^ or Fisher exact test for categorical variables and Mann–Whitney *U* or *t*-test for continuous variables were used. Survival analyses were calculated with the Kaplan–Meier method and log-rank test. To determine the effect of surgery on OS, Cox proportional hazards regression analysis was used. To prevent overfitting in the multivariable analysis, a propensity score for surgery was implemented as covariable. The propensity score was calculated by logistic regression including variables that significantly influenced OS only or both OS and surgery [26, 27]. Statistical significance was defined as a two-sided *p* value below 0.05. Statistical analysis was performed with IBM SPSS Statistics for Windows version 25 (IBM Corp., Armonk, NY) and R 3.3.3 open-source.

## Results

### PRRT

Between 2000 and 2019, 49 panNEN patients were treated with PRRT in a neo-adjuvant or downstaging setting at our centre. Baseline characteristics are summarized in Table 1. In two patients, histological examination was inconclusive and the diagnosis was established in the MDT based on clinical course of the disease and imaging. Revision of the tumour grade was not possible in 11 patients due to unavailable samples or cytological samples only. One patient had a neuroendocrine carcinoma, which showed the highest uptake score on the ^111^In-DTPA-octreotide scan. Overall, 29 of 49 patients (59%) had metastatic disease at baseline, predominantly in the liver and lymph nodes.

**Table 1:** Baseline clinical and tumour characteristics

Characteristics	All patients *n* = 49	PRRT + surgery *n* = 26	PRRT-only^a^ *n* = 23	*p* value
Age, mean (SD), years	56.1 (11.8)	54.7 (9.7)	57.7 (13.8)	0.39
Male sex, no. (%)	25 (51%)	12 (46%)	13 (57%)	0.47
BMI, median (IQR), kg/m^2^	25.1 (22.5–27.1)	25.0 (21.9–26.6)	25.2 (22.5–28.1)	0.42
Karnofsky performance score, median (IQR)	100 (90–100)	100 (90–100)	90 (90–100)	0.25
Time since diagnosis, median (IQR), months	3.8 (2.9–5.8)	3.2 (2.1–5.0)	4.4 (3.5–7.1)	0.03
Tumour grade, no. (%)^b^				<0.0001
Grade 1 NET	25 (51%)	20 (77%)	5 (22%)	
Grade 2 NET	12 (24%)	4 (15%)	8 (35%)	
Grade 3 NET	2 (4%)	1 (4%)	1 (4%)	
NEC	1 (2%)	1 (4%)	0	
No grade available	7 (14%)	0	7 (30%)	
No pathological diagnosis	2 (4%)	0	2 (9%)	
Ki67%^c^, median (IQR)	1 (1–7)	1 (1–3)	7 (1–15)	0.01
Functional, no. (%)^d^	3 (6%)	2 (8%)	1 (4%)	1.0
Progression before PRRT, no. (%)	4 (8%)	1 (4%)	3 (13%)	0.33
Location of tumour in pancreas, no. (%)				0.85
Head	26 (53%)	13 (50%)	13 (57%)	
Body	6 (12%)	4 (15%)	2 (9%)	
Tail	10 (20%)	6 (23%)	4 (17%)	
Multiple regions	7 (14%)	3 (12%)	4 (17%)	
Pancreatic tumour size, median (IQR), mm	68 (50–95)	69 (47–91)	65 (53–100)	0.54
Lymph node metastases (N1), no. (%)	15 (31%)	8 (31%)	7 (30%)	0.98
Liver metastases (M1a), no. (%)	20 (41%)	12 (46%)	8 (35%)	0.42
≥ 3 liver metastases, no. (%)	5 (10%)	4 (15%)	1 (4%)	0.35
Prior treatment, no. (%)				
Somatostatin analogue	5 (10%)	3 (12%)	2 (9%)	1.0
Surgery^e^	3 (6%)	3 (12%)	0	0.24
Chemotherapy	1 (2%)	0	1 (4%)	0.47
Liver embolization	1 (2%)	0	1 (4%)	0.47
Uptake on ^111^In-DTPA-octreotide scan, no. (%)				0.13
Equal to liver	3 (6%)	0	3 (13%)	
Higher than liver	24 (49%)	12 (46%)	12 (52%)	
Higher than kidneys/spleen	17 (35%)	11 (42%)	6 (26%)	
^68^ Ga-DOTATATE PET-CT, no. (%)	5 (10%)	3 (12%)	2 (9%)	1.0
Chromogranin A, median (IQR), µg/L	157 (72–682)	134 (69–896)	184 (72–553)	0.88

*p* values for differences between the PRRT + surgery group and PRRT-only group were calculated with *t*-test, Mann–Whitney *U*, χ^2^ test, or Fisher’s exact test

Abbreviations: *BMI*, body mass index; *NET*, neuroendocrine tumour; *NEC*, neuroendocrine carcinoma

^a^Reasons for not performing surgery were unresectability of the panNEN or metastases (*n* = 15) and high-risk surgical resection (*n* = 4), as judged by the treating physicians, as well as progression or death (*n* = 2), patient request (*n* = 1), and unknown (*n* = 1)

^b^When no biopsy or no Ki67% obtained prior to PRRT was available, the surgical resection specimen or biopsy after PRRT was used for the Ki-67 antibody staining

^c^In the 39 patients for whom Ki67% was available

^d^Glucagonoma, VIPoma, and parathyroid hormone related protein production

^e^Surgical interventions prior to PRRT consisted of a gastroenterostomy in two patients and a bilateral ovariectomy in one patient

Eleven (22%) patients did not receive the intended activity of 29.6 GBq, mainly because of bone marrow toxicity (Table 2). In 23 (47%) patients, short-term grade 3/4 bone marrow toxicity occurred, consisting of only lymphocytopenia in 18 (78%) of these patients. Grade 3 liver toxicity occurred in one patient. One patient died after the first cycle because of sepsis, unrelated to PRRT due to the absence of neutropenia.

**Table 2:** Details of peptide receptor radionuclide therapy

Characteristics	All patients *n* = 49	PRRT + surgery *n* = 26	PRRT-only *n* = 23	*p* value
Cumulative PRRT activity, median (range), GBq	29.8 (3.9–30.6)	29.9 (22.3–30.6)	29.7 (3.9–30.4)	0.03
Cumulative PRRT activity, GBq, no. (%)				
29.2–30.6	38 (78%)	24 (92%)	14 (61%)	0.06
25.8–26.6	4 (8%)	1 (4%)	3 (13%)	
18.5–22.7	5 (10%)	1 (4%)	4 (17%)	
3.9–11.2	2 (4%)	0	2 (9%)	
No. of cycles of PRRT, median (range)	4 (1–7)	4 (3–7)	4 (1–5)	0.11
Reasons for dose adjustment, no.				
Bone marrow toxicity	6	2	4	
Thrombocytopenia and patient request	1	0	1	
Recurrent cholangitis	1	0	1	
Death after first cycle of PRRT	1	0	1	
Maximum kidney dose	1	0	1	
Unknown	1	0	1	

*p* values for differences between the PRRT + surgery group and PRRT-only group were calculated with Mann–Whitney *U* or Fisher’s exact test

Abbreviations: *PRRT*, peptide receptor radionuclide therapy; *GBq*, gigabecquerel

### Response to PRRT

The best response according to RECIST 1.1 occurred at a median of 8.2 months (IQR 7.6–9.0) after the start of PRRT: partial response (PR) in 22 (45%) patients, stable disease (SD) in 24 (49%) patients, and progressive disease in two (4%) patients. The largest decrease in the panNEN size was observed at a median of 11.7 months (IQR 8.8–16.0) after the start of PRRT, where the mean change was a decrease of 26.0% ± 19.0.

### Surgical outcomes

Following the completion of PRRT, 21 patients underwent surgery of the panNEN and five patients underwent pancreatic surgery combined with liver-directed therapy (PRRT + surgery group).

Patients in the PRRT + surgery group had similar baseline characteristics compared to those who did not undergo pancreatic surgery after PRRT (PRRT-only group) with the exception of more grade 1 tumours and shorter time from diagnosis until the start of PRRT (Table 1). The PRRT-only group more often received a lower than the intended activity of PRRT (39% vs. 8%, *p* = 0.008), accompanied by a lower median overall activity (Table 2). Radiological response was more favourable for the PRRT + surgery group (62% PR, 38% SD) versus the PRRT-only group (26% PR, 61% SD, *p* = 0.02).

Details of the surgical procedures and outcomes are presented in Table 3. Resection with or without reconstruction was performed of the SMV (*n* = 3), PV (*n* = 2), and CA (Appleby procedure, *n* = 1). Thrombectomy of the PV was required in one patient. Resection margins were free of tumour cells in 19 (73%) patients (R0 resection).

**Table 3:** Surgical and non-surgical interventions and outcomes in the PRRT + surgery group

Characteristics	*n* = 26
Time since start of PRRT, median (IQR), months	14.8 (12.2–18.3)
Time since end of PRRT, median (IQR), months	8.4 (6.1–11.6)
Type of surgery, no. (%)	
Pancreaticoduodenectomy (Whipple)	10 (38%)
Distal pancreatectomy with splenectomy	10 (38%)
Pylorus-preserving pancreaticoduodenectomy	6 (23%)
Vascular resection/reconstruction, no. (%)	6 (23%)
Treatment of liver metastases^a^, no. (%)	5 (19%)
Postoperative complications^b^, no. (%)	17 (65%)
Grade II	
Infection	5
Thromboembolic	2
Delayed gastric emptying	1
De novo diabetes mellitus	1
Pancreatitis	1
Total parenteral nutrition for insufficient oral intake	1
Grade IIIa	
Leakage	7
Infection	2
Grade IIIb	
Venous bypass occlusion	1
Stenosis hepaticojejunostomy (IIIb-d)	1
Enterocutaneous fistula of the transverse colon	1
Stomach perforation	1
Grade IVa	
Haemodynamic instability due to perioperative blood loss	1
Grade V	
Haemorrhagic shock caused by a rupture of the portal vein during surgery and a mesenteric bleed after surgery	1
Resection margins, no. (%)	
R0 resection	19 (73%)
R1 resection	7 (27%)
No. resected lymph nodes, mean (SD)	7 (5)
No. positive lymph nodes^c^, median (IQR)	0 (0–3)
Lymphovascular invasion, no. (%)	
Negative	17 (65%)
Positive	9 (35%)
Perineural invasion, no. (%)	
Negative	19 (73%)
Positive	7 (27%)
Ki67%, median (IQR)	1 (1–4)
Percentage of fibrosis, mean (SD)	52 (25)
Homogeneous, no. (%)	9 (38%)
Heterogeneous, no. (%)	15 (63%)
Presence of necrosis, no. (%)	2 (8%)
Percentage of viable tumour, mean (SD)	47 (24)
Percentage of SSTR2a-positive tumour cells, median (IQR)	100 (58–100)

Abbreviations: *SSTR2a*, somatostatin receptor subtype 2a

^a^Treatment of liver metastases included a single wedge resection (*n* = 3), radiofrequent ablation (*n* = 2), multiple wedge resections (*n* = 1), and microwave ablation (*n* = 1)

^b^According to the Clavien-Dindo classification of surgical complications [54], including grade ≥ II

^c^Considering only the 24 patients in whom at least one lymph node was resected

After surgery with or without treatment of metastases, there was no evidence of tumour presence on the first postoperative radiological or SSTR imaging in 22 (85%) patients, including one patient whose liver metastasis was resected 6 months after pancreatic surgery.

Reassessment of the surgical resection specimen was available for 24 patients. Fibrosis was present in all samples and comprised on average 52% of the tumour. In the patients with PR after PRRT, 59% ± 25 of the tumours consisted of fibrosis compared to 41% ± 21 in the patients with SD (*p* = 0.08).

### Retrospective radiological review

Tumour contact of the SMA, CA, CHA, SMV, and PV by the panNENs is presented in Table 4. In those patients with TVI before PRRT, relevant downstaging of the involvement was observed in 38% of patients overall: 10 out of 21 (48%) patients from the PRRT + surgery group and five out of 18 (28%) of PRRT-only patients (*p* = 0.20).

**Table 4:** Radiological vascular involvement before and after PRRT

TVI, no (%)	PRRT + surgery, *n* = 26				PRRT-only, *n* = 19^a^			
	Baseline	After treatment with PRRT			Baseline	After treatment with PRRT		
		No change	Decrease	Increase		No change	Decrease	Increase
No TVI	5 (19%)	5/5 (100%)	0/5 (0%)	0/5 (0%)	1 (5%)	1/1 (100%)	0/1 (0%)	0/1 (0%)
Vein^b^ only								
≤ 180°	7 (27%)	6/7 (86%)	1/7 (14%)	0/7 (0%)	2 (11%)	1/2 (50%)	1/2 (50%)	0/2 (0%)
> 180°^c^	1 (4%)	1/1 (100%)	0/1 (0%)	0/1 (0%)	3 (16%)	1/3 (33%)	0/3 (0%)	2/3 (67%)
Artery^d^ and vein^b^								
artery ≤ 180° vein ≤ 180°	3 (12%)	1/3 (33%)	2/3 (67%)	0/3 (0%)	1 (5%)	1/1 (100%)	0/1 (0%)	0/1 (0%)
artery ≤ 180° vein > 180°^c^	5 (19%)	2/5 (40%)	3/5 (60%)	0/5 (0%)	5 (26%)	3/5 (60%)	1/5 (20%)	1/5 (20%)
artery > 180° vein ≤ 180°	2 (8%)	1/2 (50%)	1/2 (50%)	0/2 (0%)	1 (5%)	1/1 (100%)	0/1 (0%)	0/1 (0%)
artery > 180° vein > 180°^c^	3 (12%)	0/3 (0%)	3/3 (100%)	0/3 (0%)	6 (32%)	3/6 (50%)	3/6 (50%)	0/6 (0%)
Total	26	16/26 (62%)	10/26 (38%)	0/26 (0%)	19	11/19 (58%)	5/19 (26%)	3/19 (16%)

*p* values for differences between the PRRT + surgery group and PRRT-only group were calculated with Fisher’s exact test; all *p* values were > 0.05

Abbreviations: *TVI*, tumour-vessel interface

At baseline, a median of 3 (range 1–5) vessels were involved in the patients with TVI: the SMV in 33 patients, PV in 28 patients, CHA in 21 patients, SMA in 12 patients, and the CA in five patients. Other involved vessels not included in this table were the splenic vein (*n* = 25), splenic artery (*n* = 23), gastroduodenal artery (*n* = 21), inferior vena cava (*n* = 3), jejunal arteries (*n* = 3), and jejunal veins (*n* = 2)

It was considered a relevant decrease or increase in TVI if a patient had a respectively lower or higher involvement category after PRRT compared to baseline

^a^Four PRRT-only patients were excluded due to unavailable scans

^b^Superior mesenteric vein and/or portal vein

^c^TVI > 180° or occlusion due to tumour ingrowth or thrombus

^d^Superior mesenteric artery and/or celiac axis and/or common hepatic artery

After PRRT, six (23%) patients in the PRRT + surgery group and three (16%) patients in the PRRT-only group displayed no TVI, whereas a median of 2 (range 1–5) vessels were involved in the other patients.

More than 180° encasement or occlusion of at least one vascular structure was observed in 11 (42%) PRRT + surgery patients and in 15 (79%) PRRT-only patients (*p* = 0.01) before PRRT, and in seven (27%) and in 14 (74%) patients after PRRT, respectively (*p* = 0.002).

Organ invasion (stomach, duodenum, spleen, liver, adrenal) was present in 10 (38%) patients in the PRRT + surgery group and in 11 (58%) patients in the PRRT-only group at baseline (*p* = 0.20). After PRRT, the presence of organ invasion decreased to five (19%) and nine (47%) patients, respectively (*p* = 0.04).

Disappearance of all detectable liver metastases occurred in five of the 12 patients in the PRRT + surgery group, obviating the need for liver-directed treatment, and in none of the eight patients in the PRRT-only group (*p* = 0.06).

### Survival outcomes

**Fig. 1:**
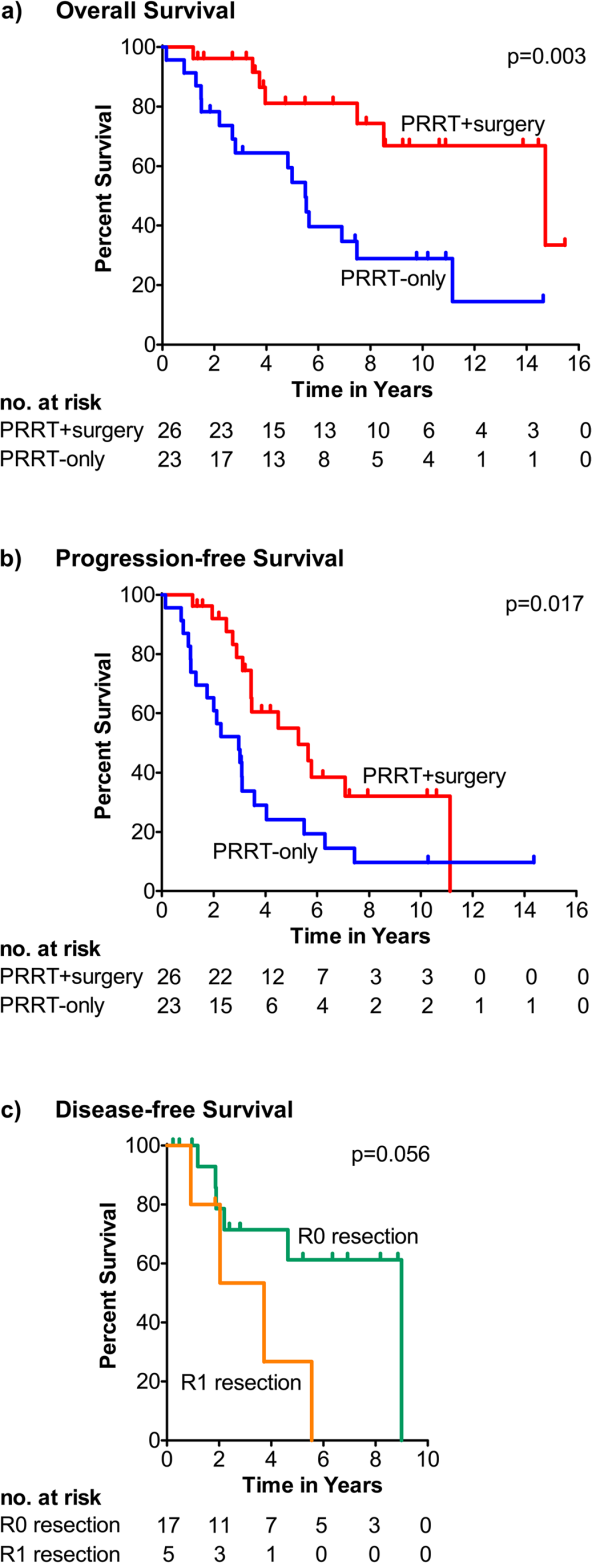
Survival analysis. Kaplan–Meier curves of overall survival (**a**) and progression-free survival (**b**), measured from the first cycle of PRRT, for the patients with locally advanced or oligometastatic panNEN treated with PRRT. **c** Disease-free survival, measured from the date of surgery, for the patients with no evidence of disease on the first radiological or somatostatin receptor imaging postoperatively, stratified according to the resection margins. *p* values were calculated using the log-rank test

Seven patients in the PRRT + surgery group and 16 patients in the PRRT-only group died during a median follow-up period of 5.5 years (IQR 2.8–9.4). Two patients in this study cohort developed fatal haematological malignancies 2 and 5 years after completion of the treatment, which was judged as probably related to PRRT. The median OS in the entire cohort was 8.5 years (95% CI 4.5–12.5). Patients in the PRRT + surgery group had a significantly longer OS of 14.7 years (95% CI 5.9–23.6) compared to 5.5 years (95% CI 4.5–6.5) for the PRRT-only patients (*p* = 0.003, Fig. 1a). In the univariable Cox proportional hazards analysis for OS, the hazard ratio (HR) of surgery was 0.264 (95% CI 0.103–0.678, *p* = 0.006). A propensity score was created with the cumulative activity, grade 1 NET versus other or no available grade, Karnofsky performance score (KPS), and the panNEN size post-PRRT (Table 5). In the multivariable analysis including surgery and the propensity score, the HR of surgery was 0.629 (95% CI 0.190–2.086, *p* = 0.449) and the HR of the propensity score was 0.125 (95% CI 0.017–0.908, *p* = 0.040).

**Table 5:** Univariable analyses for all patients (*n* = 49)

Variables^a^	Binary logistic regression for surgery		Cox proportional hazards analysis for overall survival	
	OR (95% CI)	*p* value	HR (95% CI)	*p* value
Karnofsky performance score	1.061 (0.980–1.149)	0.145	0.934 (0.890–0.980)	0.005
Grade 1 NET	12.000 (3.121–46.144)	< 0.001	0.264 (0.106–0.661)	0.004
Cumulative PRRT activity, GBq	1.319 (1.004–1.732)	0.047	0.895 (0.826–0.970)	0.007
Partial response	4.533 (1.337–15.368)	0.015	0.413 (0.165–1.036)	0.059
PanNEN size post-PRRT, mm^b^	0.974 (0.949–1.000)	0.050	1.019 (1.002–1.036)	0.027

Abbreviations: *OR*, odds ratio; *HR*, hazard ratio; *CI*, confidence interval; TVI, tumour-vessel interface; *PRRT*, peptide receptor radionuclide therapy; *GBq*, gigabecquerel

^a^Only variables with a *p* < 0.05 are presented. Age, sex, presence of metastases, number of involved vessels post-PRRT, no TVI vs. ≤ 180° TVI vs. > 180° of TVI post-PRRT, no TVI vs. venous and/or arterial TVI post-PRRT, and organ invasion post-PRRT were not statistically significant

^b^Two patients were excluded because the post-PRRT scan was not available

Radiological disease progression occurred in 13 patients in the PRRT + surgery group and 15 patients in the PRRT-only group during follow-up. In the PRRT + surgery group, the initial progression consisted of new liver metastases and/or progression of present liver metastases (*n* = 8), local recurrence (*n* = 3), new bone metastases (*n* = 1), and new bone and liver metastases (*n* = 1). In the PRRT + surgery group, the initial progression occurred in the primary pancreatic tumour (*n* = 7), in the liver (*n* = 6), and in both the pancreas and liver (*n* = 2). Clinical disease progression was observed in an additional three PRRT-only patients. The median PFS was 3.0 years (95% CI 1.6–4.4) for the PRRT-only group and 5.3 years (95% CI 2.4–8.1) for the PRRT + surgery group (*p* = 0.02, Fig. 1b).

In 10 of the 22 (45%) patients who had no evidence of disease on the first radiological or SSTR imaging postoperatively, disease recurred and resulted in a median DFS of 5.5 years (95% CI 2.6–8.5). The median DFS in the subgroup of 17 patients with an R0 resection was 9.0 years (95% CI not defined) compared to 3.7 years (95% CI 1.4–6.0) for the five patients with an R1 resection (*p* = 0.056, Fig. 1c).

## Discussion

In our series of 49 patients with a locally advanced and/or oligometastatic panNEN, induction PRRT resulted in an average 26% decrease in the panNEN size and downstaging of the vascular involvement in 38% of the patients with TVI. After PRRT, 26 patients underwent surgery with curative intent, which was accompanied by favourable long-term outcomes.

A considerable group of panNEN patients is not eligible for upfront curative surgery, since 16% of these patients present with regionally advanced disease and 44% with metastatic disease [1]. Moreover, major vascular structures and adjacent organs are involved in up to 17% [28] and 19% [10], respectively, of the panNEN patients who were evaluated for curative surgical resection. Despite its associated risk of morbidity and mortality [4, 29–32], pancreatic surgery with curative intent has a favourable effect on survival in panNEN patients [2, 3, 33, 34] and is therefore recommended in the guidelines [6, 7, 35]. Unfortunately, recurrence rates after surgery reach up to 69%, depending on the tumour grade, stage, size, and the duration of the studies’ follow-up [9, 12, 21, 30, 36–40], limiting the prognosis of these patients. In contrary to locally advanced pancreatic adenocarcinoma [41], no systemic induction treatment strategy is advocated by the guidelines for locally advanced or oligometastatic panNENs before surgery since the high-quality evidence supporting induction or neo-adjuvant treatment with PRRT or other agents is lacking.

^117^Lu-DOTATATE results in an OR in up to 57% in advanced panNEN patients [15–18] and has, therefore, potency as induction therapy for downstaging as well as improving long-term outcomes. In our series, induction PRRT resulted in PR in 45% of all patients. This appears comparable to the PR rate of 43% of induction therapy with capecitabine and temozolomide [42]. Capecitabine with temozolomide could therefore also be a potential induction strategy; however, details on vascular downstaging were not provided. PRRT led to downstaging of vascular and organ invasion in 38% and 33% of the patients in our cohort, respectively, ultimately allowing for surgical resection in 26 patients. Preoperative chemotherapy with fluorouracil, doxorubicin, and streptozocin resulted in PR in 7% of all patients and vascular downstaging in 24% of the patients with TVI [24]. Following PRRT, successfully operated patients had a higher rate of downstaging TVI of > 180° and less organ invasion.

In this study, in only six patients concomitant vascular resection with or without reconstruction was needed. In a series of 42 panNEN patients with vascular involvement on preoperative imaging, vascular involvement was detected perioperative in 15 patients and vascular reconstruction had to be performed in nine patients [28]. In another series, 25 of 99 locally advanced panNEN patients had vascular involvement on preoperative imaging and vascular reconstruction was required in 17 patients [12]. One cause of diminished vascular tumour invasion and potential underestimation of response is the presence of fibrosis after PRRT, which comprised approximately half of the tumour volumes and appeared to be more present in patients with a better response to PRRT.

Importantly, the criteria for assessing locoregional resectability of pancreatic malignancies remain an area of debate without dedicated surgical criteria for panNEN [41, 43]. The NANETS recommended to not consider isolated major vascular involvement with or without venous tumour thrombus as an absolute contraindication to surgical panNEN resection [35]. A limited number of studies illustrated that panNEN surgery with vascular resection or reconstruction with/without adjacent organ resection was feasible with encouraging long-term survival. However, it has also been reported that these concurrent resections were associated with worse outcomes regarding morbidity, recurrence-free survival, and/or OS compared to pancreatic surgery without vascular or organ resection [10–14, 28, 44, 45], underlining the need of an induction and neo-adjuvant strategy.

The median OS was 14.7 years in the PRRT + surgery group, which is considerably longer than the 5.5 years in the PRRT-only group as well as the reported survival outcome of 7.8 years for regionally advanced panNEN [46]. Despite the surgical complications and PRRT-related toxicity, we observed long-term survival in patients who were previously ineligible for surgery or needed high-risk surgery. However, there were baseline differences between the PRRT + surgery and PRRT-only group that needed to be taken into account. In the survival analysis, surgery reduced the risk of all-cause death, but this was not statistically significant after adjustment with a propensity score for surgery including grade, KPS, panNEN size, and cumulative activity. This likely reflects that the superior prognostic outcome in these locally advanced panNEN patients is primarily linked to specific baseline features and response to PRRT rather than the presence of a surgical resection.

In 10 of the 22 patients who had no detectable disease on imaging after surgery, the disease recurred after a median of 5.5 years. Given this long interval in our highly selected patient group with elevated risk of recurrence, PRRT could have produced a neo-adjuvant effect. However, it needs to be addressed that the ^68^ Ga-DOTATATE PET-CT scan was not yet available for the majority of our patients and we did not routinely perform MRI scans of the liver, both imaging modalities that are superior in detecting liver metastases compared to the ^111^In-DTPA-octreotide scintigraphy [47] and CT-scan [48], respectively. This could have influenced the detection of recurrence as well as the response assessment of the liver metastases that disappeared after PRRT in five of the 12 PRRT + surgery patients.

Nonetheless, our results appear to be in line with the study from Partelli et al. on the outcomes of PRRT for resectable or potentially resectable panNEN with features associated with a high recurrence risk, albeit with lower TVI than in our study. In this study, 23 patients underwent neo-adjuvant PRRT followed by resection and 23 patients underwent upfront surgery. After PRRT, significantly fewer patients had SMV/PV invasion (48% vs. 18%). Moreover, the authors described that neo-adjuvant PRRT could decrease the risk of postoperative pancreatic fistula and the rate of lymph node metastases. However, the median PFS was equal at 4.3 years in the neo-adjuvant PRRT group versus 3.1 years in the upfront surgery group. Only in the subgroup of patients with an R0 resection, the PFS was significantly longer after neo-adjuvant PRRT [21]. Ideally, the PRRT + surgery group would have also been compared to a surgery-only group to investigate the neo-adjuvant effect. However, a large subset of our patients had, prior to PRRT, locally advanced disease for which resection was not deemed feasible or safe. Comparing our cohort to a group of patients who are eligible for upfront surgery with curative intent would therefore risk major selection bias towards patients with less extensive disease and thus better outcomes.

Our study population is relatively small, which makes type two errors more likely to occur. However, panNEN in general, and panNEN with a tumour stage limited to local disease at diagnosis specifically, are rare. To our knowledge, this is the largest patient group described in the literature. Another limitation of this study is its retrospective design for patient inclusion and the risk of bias in patient selection﻿ for early PRRT and/or for surgery. Long-term outcomes from a prospective trial with implementation of international inclusion criteria for resectability and indication for downstaging of panNEN are needed, but are, given the rarity and indolent nature of panNEN, difficult to obtain. Furthermore, future research should assess which selection criteria should be applied for induction PRRT. These could also include new potential prognostic factors for recurrence such as loss of ATRX/DAXX, ALT-positivity [49], and α-cell origin [50], as well as predictors of response to PRRT, such as the PRRT predictive quotient [51], or of recurrence, such as the NETest [52, 53].

In conclusion, PRRT can be a viable induction treatment option for patients with locally advanced and/or oligometastatic panNENs who are not eligible for upfront curative surgery. Its potency in securing long-term survival after surgery in this high-risk patient group also suggests a neo-adjuvant effect. Expert MDT discussions are essential in case of locoregionally advanced panNEN to select patients who may benefit from induction PRRT before surgery.

## Data Availability

The datasets generated during and/or analysed during the current study are not publicly available, but are available from the corresponding author on reasonable request.
